# Editorial: Recent advances in diagnosis and treatment of congenital adrenal hyperplasia due to 21-hydroxylase deficiency

**DOI:** 10.3389/fendo.2023.1332962

**Published:** 2023-11-22

**Authors:** Semra Çaglar Çetinkaya

**Affiliations:** ^1^ University of Health Sciences (Turkey), Istanbul, Türkiye; ^2^ Dr Sami Ulus Child Health and Diseases Training and Research Hospital, Ankara, Türkiye

**Keywords:** 21 hydroxylase deficiency, follow-up, treatment, new therapies, comorbidities

This special edition Research Topic was designed to evaluate all aspects of congenital adrenal hyperplasia due to 21-hydroxylase deficiency (21OHD). Congenital adrenal hyperplasia (CAH) due to 21OHD is the most common type of CAH (90-99% frequency) (1). Depending on the degree of enzyme deficiency, signs of glucocorticoid and/or mineralocorticoid deficiency are observed. The insufficient glucocorticoid and mineralocorticoid hormones are replaced in the treatment. The dose adjustment is important in the treatment. Excess doses cause hypercortisolism, while insufficient doses cause hyperandrogenism. Both hypercortisolism and hyperandrogenism can result in negative cardiometabolic outcomes, for example, and thus, CAH is associated with long-term health risks (2).

## The correlation of genotype and phenotype in CAH

Tang et al. investigated the relationship between genotype and phenotype in 15 individuals diagnosed with CAH due to 21OHD from three unrelated families and showed dramatically different phenotypes in the three probands with different compound heterozygous pathological variants in *CYP21A2.* The authors stated that genetic analysis could help the etiologic diagnosis, especially for atypical 21OHD patients, because of wide-spectrum residual enzyme activity.

Zhao et al. focused on another aspect of the genotype and phenotype relationship in CAH cases with 21OHD. Observing that the genotype harboring the P31L promoter variant in *CYP21A2* has a simple virilization phenotype at a rate of 57.1%, this study drew attention to the importance of elaborating the phenotype–genotype relationship in CAH cases.

## The diagnostic and monitoring methods of CAH

There have been changes in the diagnostic process of CAH in the last 15 years. Nakhleh et al. investigated the value of 17 hydroxy-progesterone (17OHP) measured by ELISA and RIA in diagnosing non-classical CAH with the cosyntropin test in their nearly 20-year-old archives. Although 17OHP has been a marker used in the diagnosis, screening, and monitoring of CAH for 40 years, its reliability is poor. Therefore, there is a need to develop new techniques that can measure adrenal androgens and their precursors. In their mini-review, de Hora et al. emphasized the advantages of the liquid chromatography–tandem mass spectrometry method for this purpose. In particular, that the adrenal steroidogenesis backdoor pathway is more active in newborns diagnosed with CAH and that oxygenated androgens are high in untreated CAH cases have made it important to analyze androgenic steroids and their precursors by the liquid chromatography–tandem mass spectrometry method. Especially, 21-deoxycortisol has emerged as a marker of 21OHD.

## The follow-up of CAH

Itonaga et al. compared the first morning urinary pregnanetriol with 17OHP values at various times during the day for the biochemical monitoring of 21OHD. They showed that the first morning pregnanetriol before morning medication correlated well with 17OHP and could be a good marker (more practical and useful) for monitoring 21OHD.

## The comorbidities of CAH

Auchus et al. conducted a survey study in which they investigated experts’ opinions on treatment practices and unmet needs in adults with classical CAH (a modified Delphi consensus study). In this study, all panelists stated that glucocorticoid-related comorbidities are difficult to treat in CAH cases and that new treatments are important to prevent them.

Lim et al. compared Korean adults with CAH (71 men-mean age: 27 years, 93 women-mean age: 28 years) with age- and sex-matched healthy controls in terms of some long-term health risks and comorbidities [obesity, testicular adrenal rest tumors (TARTs), menstrual irregularity]. In this study, the researchers found high waist circumference and blood pressure in CAH adults according to the control group. They reported that the TARTs frequency in men was 58.1%, and the irregular menstruations’ frequency in women was 57.1% (both genders had the same treatment regimens and hormonal status). They found a 2.7-fold increased risk for hypertension in men with CAH and a 2.0-fold increased risk for women with obesity and CAH. They showed higher adrenal limb thicknesses (men) and 17OHP and dehydroepiandrosterone sulfate levels (women) in obese CAH cases. The authors pointed out that poor metabolic control in CAH patients increases the risk of metabolic morbidity.

In the study of Saho et al., the prevalence of TART (all salt-wasting form and poor metabolic control) in Slovakian patients with CAH was reported to be 29%. Harasymiw et al. investigated the prevalence of depressive and anxiety disorders in CAH individuals (4-25 y) and non-CAH individuals in the United States. They found a higher adjusted prevalence ratio (aPR) for depressive disorders, anxiety disorders, and antidepressant prescriptions in patients with CAH compared to the controls. The metabolic risks and comorbidities should be investigated in CAH patients. Checklists can be created to facilitate the routine monitoring of patients with CAH.

Shafaay et al. assessed the clinical characteristics and quality of life (QoL) (main evaluation domains: mobility, self-care, usual activities, pain/discomfort, and anxiety/depression) of pediatric and adult CAH patients. They found that CAH patients had lower QoL scores in all domains compared to the healthy control group (in particular, approximately half of those patients the in pain/discomfort and anxiety/depression domains). In addition, this study also stated that decreased mobility is the most important risk factor for lower QoL in obese CAH patients. They recommended a multidisciplinary team approach, pre-marital screening, and the implementation of awareness programs for CAH cases, especially in highly consanguineous communities.

## The new treatment strategies in CAH

In their study, Khattab and Charlton showed that testicular testosterone levels increased with tildacerfont treatment, an oral active corticotrophin-releasing factor type 1 (CRF1) receptor antagonist, at different doses in patients with uncontrolled CAH. They emphasized that this drug is a new treatment option for cases developing TART and azoospermia and that further studies are needed.

In this Research Topic, the diagnosis, follow-up and treatment processes, and possible comorbidities of CAH cases with different levels of enzyme activities due to 21OHD were evaluated. New methods such as the liquid chromatography–tandem mass spectrometry method for hormonal profile analysis of CAH cases, new monitoring markers such as first monitoring urinary pregnanetriol, neglected problems such as pain/discomfort and anxiety/depression, and new treatment options such as CRF1 receptor antagonist have been presented. As a Research Topic, CAH is a broad and complex field on which more study needs to be performed.

## Author contributions

SÇÇ: Writing – original draft.

## References

[1] Claahsen-van der Grinten HL Speiser PW Ahmed SF Arlt W Auchus RJ Falhammar H. Congenital adrenal hyperplasia-current insights in pathophysiology, diagnostics, and management. Endocr Rev (2022) 43(1):91–159. doi: 10.1210/endrev/bnab016 33961029 PMC8755999

[2] Nordenström A Lajic S Falhammar H. Long-term outcomes of congenital adrenal hyperplasia. Endocrinol Metab (Seoul). (2022) 37(4):587–98. doi: 10.3803/EnM.2022.1528 PMC9449109 35799332

